# Antibody against Extracellular Vaccinia Virus (EV) Protects Mice through Complement and Fc Receptors

**DOI:** 10.1371/journal.pone.0020597

**Published:** 2011-06-08

**Authors:** Matthew E. Cohen, Yuhong Xiao, Roselyn J. Eisenberg, Gary H. Cohen, Stuart N. Isaacs

**Affiliations:** 1 Division of Infectious Diseases, Department of Medicine, School of Medicine, University of Pennsylvania, Philadelphia, Pennsylvania, United States of America; 2 Department of Microbiology, School of Veterinary Medicine, University of Pennsylvania, Philadelphia, Pennsylvania, United States of America; 3 Department of Microbiology, School of Dental Medicine, University of Pennsylvania, Philadelphia, Pennsylvania, United States of America; University of Georgia, United States of America

## Abstract

Protein-based subunit smallpox vaccines have shown their potential as effective alternatives to live virus vaccines in animal model challenge studies. We vaccinated mice with combinations of three different vaccinia virus (VACV) proteins (A33, B5, L1) and examined how the combined antibody responses to these proteins cooperate to effectively neutralize the extracellular virus (EV) infectious form of VACV. Antibodies against these targets were generated in the presence or absence of CpG adjuvant so that Th1-biased antibody responses could be compared to Th2-biased responses to the proteins with aluminum hydroxide alone, specifically with interest in looking at the ability of anti-B5 and anti-A33 polyclonal antibodies (pAb) to utilize complement-mediated neutralization in vitro. We found that neutralization of EV by anti-A33 or anti-B5 pAb can be enhanced in the presence of complement if Th1-biased antibody (IgG2a) is generated. Mechanistic differences found for complement-mediated neutralization showed that anti-A33 antibodies likely result in virolysis, while anti-B5 antibodies with complement can neutralize by opsonization (coating). In vivo studies found that mice lacking the C3 protein of complement were less protected than wild-type mice after passive transfer of anti-B5 pAb or vaccination with B5. Passive transfer of anti-B5 pAb or monoclonal antibody into mice lacking Fc receptors (FcRs) found that FcRs were also important in mediating protection. These results demonstrate that both complement and FcRs are important effector mechanisms for antibody-mediated protection from VACV challenge in mice.

## Introduction

In the 1970s, the World Health Organization led a successful campaign to eradicate smallpox using live vaccinia virus (VACV) vaccines [1]. However, recent concern over the intentional or accidental release of variola virus has led some of the world's nations to stockpile live VACV vaccines [2]–[4]. With the risk of variola virus release minimal, concerns regarding live VACV vaccine's rare but serious side effects and many contraindications [5]–[7] have led to the pursuit of safer smallpox vaccine strategies [8]–[10]. Modified vaccinia virus Ankara (MVA), a highly attenuated VACV-derived vaccine, has been under development and will likely soon become a safer alternative [11], [12]. However, subunit vaccination is an approach that does not rely on production of a virus. We evaluated the efficacy and mechanism by which a protein-based subunit vaccine can protect against orthopoxvirus infection.

After vaccination, protection from orthopoxvirus disease heavily depends on antibody responses in animal models [13]–[15] and humans [16], [17]. Many of the responses are directed against viral surface proteins on the two virion forms, mature virus (MV) and extracellular virus (EV). The MV form is the most abundant virion form in infected cells [18] and is believed to mediate spread between hosts. The EV form mediates dissemination within an infected host [19]–[22]. The MV form contains a large set of surface proteins, while the EV form contains an extra membrane and an additional, unique subset of surface proteins. Antibody against certain proteins of either form can be partially protective, such as L1 on MV [23]–[27] and B5 or A33 on EV [15], [23], [26], [28]–[30], though optimal protection is seen when antibodies are directed against both forms [23]–[26], [31], [32]. Subunit protein vaccination including target antigens from both forms achieves protection from lethal orthopoxvirus challenge in mouse and non-human primate challenge models [23], [32]–[35]. In theory, antibody generated against the MV form would act to neutralize a portion of the initial infectious dose and antibody against the EV form could then prevent some spread of progeny virus within a host. Having these antibody responses present at the time of challenge could then allow the host time to generate additional immune responses and provide protection from lethal disease.

Serum from vaccinated animals or humans is capable of efficiently neutralizing the MV form of VACV [23], [32], [34], [36], [37]; however, direct antibody neutralization of the EV form has been suboptimal at even high concentrations of anti-EV antibody [15], [29], [38]–[41]. Therefore, understanding the mechanism by which anti-EV antibodies provide protection has been of interest. Recent mouse studies have elucidated that an IgG2a isotype monoclonal antibody (mAb) against the B5 protein called B126 can neutralize EV in the presence of complement (C') and utilizes C' to partially mediate protection in vivo [42], [43]. This evidence suggests that antibody against EV would be more effective if it was of an isotype that mediated effector functions such as activation of C' and/or Fc receptor (FcR) dependent activity (e.g. antibody dependent cellular cytotoxicity (ADCC)). Previous studies of antibody responses to protein vaccination found that formulations that included adjuvants that produced higher titers of IgG2a antibody in mice and IgG1 antibody in non-human primates were more effective at mediating protection than vaccines formulated without these adjuvants [33], [34]. This suggests that antibody with specific Fc activities might be beneficial for protection.

By utilizing a high PFU luciferase reporter EV neutralization assay, we find that polyclonal antibody responses against the EV proteins A33 and B5 utilize C' to neutralize virus in vitro, though in mechanistically different ways. These findings shed light on how differing viral proteins dictate the requirements for the host to neutralize incoming virus with C'. Additionally, we show that antibody against B5 utilizes C' and FcRs to protect mice from lethal VACV challenge. These findings add to our understanding of how antibody can protect against orthopoxvirus disease and highlights the importance of understanding antibody effector functions necessary for protection to aid in the rational design of anti-viral vaccines and therapeutic antibodies.

## Materials and Methods

### Proteins and vaccine formulations

Proteins used in the vaccine formulations were purified recombinant baculovirus-expressed proteins that were previously described [34] . Protein vaccines were prepared and used as described previously [32]. Briefly, proteins (each at 2 µg/mouse) and adjuvant(s) were prepared in sterile PBS and a final volume of 50 µL was injected intramuscularly into the hind leg of ketamine/xylazine-anesthetized mice. For vaccines adjuvanted with alum only (Alhydrogel, Accurate Chemical, Westbury, NY), formulations included the alum at 200 µg aluminum ion/mouse. For vaccines formulated with alum and CpG, the alum was at 100 µg aluminum ion/mouse and the phosphorothioate B class mouse specific CpG ODN 1826 (sequence 5′-TCC ATG ACG TTC CTG ACG TT-3′; Coley Pharmaceutical Group, now Pfizer Inc.) was used at 50 µg/mouse. On the day of vaccinations, vaccine formulations were prepared, mixed at room temperature for 2–3 hours, and loaded into 0.3 mL insulin syringes with a 29-gauge needle (Becton Dickinson).

### Mice, immunizations, and challenge

BALB/c mice and C57BL/6 mice were purchased from Charles River and Jackson Laboratory, respectively. Fc-receptor knockout mice (FcRKO) mice on the BALB/c background were purchased from Taconic Farms. Complement component C3 knockout mice (C3KO) on the C57BL/6 background (originally provided by J. D. Lambris, University of Pennsylvania) were bred at the University of Pennsylvania. Active immunizations were performed as previously described [32]. Briefly, mice were primed by intramuscular vaccination, boosted 2 weeks later, and bled 1 day prior to challenge (approximately 3 weeks after the boost) to assess successful antibody production and isotype analysis. In some instances, additional mice were terminally bled prior to challenge for serum to be used in in vitro EV neutralization assays. Passive immunizations were performed using the anti-B5 rabbit polyclonal antibody (pAb) R182 [26] (2 mg of purified total rabbit IgG /mouse) or the anti-B5 monoclonal antibody (mAb) B126 [43] (100 µg of purified mouse IgG /mouse; generously provided by Kyowa Hakko Kirin Co. and S. Crotty, La Jolla Institute for Allergy and Immunology). Antibodies, diluted in sterile saline at a final volume of 300 µL/mouse for R182 or 100 µL/mouse for B126, were injected intraperitoneally (i.p.) one day prior to challenge. Control mice were given sterile saline only. Vaccinated mice were challenged as described previously [32]. Briefly, VACV (strain WR) was grown in BSC-1 cells (ATCC® Number CCL-26™) and virus from cell lysates isolated by ultracentrifugation through two sequential 36% sucrose cushions. Three weeks after the boost protein vaccination or one day after passive immunization with antibody, mice were anaesthetized with ketamine/xylazine and challenged intranasally with a lethal dose of VACV in a total volume of 20 µL (10 µL/nostril) in sterile PBS. Challenge doses were confirmed by titering on BSC-1 cells the day of challenge and indicated in the figure legends. Mice were weighed and monitored each day and mice that reached >30% starting weight or met end point criteria were humanely euthanized. Experiments were performed under a protocol that was approved by the University of Pennsylvania Institutional Animal Care and Use Committee (IACUC), Animal Welfare Assurance number A3079-01. To minimize pain, all viral challenges and intramuscular vaccinations were performed under ketamine/xylazine anesthesia. To minimize suffering after viral challenge, mice were monitored and humanely euthanized when end point criteria were met.

### Antibody ELISA

Antibody ELISA was performed as previously described [32], [34]. Briefly, plates were coated overnight at 4°C with 0.5 µg/mL non-his tagged recombinant A33, B5, or L1 protein in bicarbonate/carbonate coating buffer. After blocking with 5% non-fat dry milk in PBS, 2-fold serial dilutions of mouse sera were added and incubated for 1.5 hrs at 37°C. After washing, HRP-conjugated rabbit anti-mouse IgG (Abcam, Cambridge, MA) secondary antibody was added at 1∶4000 in blocking buffer and incubated at 37°C for 1 hr. Color development was performed using ABTS substrate (Sigma) for 20 minutes at room temperature. The reaction was stopped using 1% SDS in distilled water. IgG isotype was assessed similarly, using HRP-conjugated rat anti-mouse IgG1 or IgG2a (BD Biosciences Pharmingen), or HRP-conjugated goat anti-mouse IgG2c (SouthernBiotech, Birmingham, AL) secondary antibody diluted 1∶1000, 1∶1000, and 1∶4000 respectively in blocking buffer and incubated for 1 hr at 37°C.

### In vivo complement depletion

C' was transiently depleted using native Cobra Venom Factor (CVF) from *Naja naja kaouthia* (Quidel Corporation) as previously described [43]. Briefly, 10 µg (∼4 units) of CVF in sterile saline (100 µL total volume) was administered i.p to mice on days −1, 2, and 5 of challenge. Complement depletion was confirmed on days 0, 3, and 6 by C3 western blotting [44], C3 ELISA [43], and CH50 assay [45], [46] (Fig. S1). We found that C' was fully depleted on days 0 and 3, but C' activity and C3 protein were at ∼50% the levels of undepleted sera on day 6 confirming previous findings of transient depletion [43]. Intranasal challenge was performed on day 0.

### EV production

RK-13 cells (ATCC® Number CCL-37™) were plated in 6-well plates 2 days prior to use and used at 100% confluency. To produce EV, RK-13 cells were infected with vaccinia IHD-JvFire [47] (generously provided by B. Moss, NIH) in serum-free MEM at MOI of 0.5. Two days after infection, supernatant was harvested, clarified by centrifugation at 450× g at 4°C, and the virus remaining in the supernatant was immediately titered in the presence of MV neutralizing monoclonal antibody 2D5 (1∶500 dilution of ascites fluid) [48] . In general, based on titering of supernatants in the presence or absence of 2D5, >80% of virus in the supernatants was EV. Clarified media was stored on ice at 4°C and used within a week of isolation. For EV expressing specific human complement regulators, the same protocol for EV preparation and titering was followed except that simian virus 40-transformed aortic rat endothelial cells (SVAREC) stably transfected with plasmids expressing human CD55 or human CD59 were used [49], [50] (a generous gift of G. L. Smith, Imperial College of London). We confirmed expression of human CD55 and human CD59 in these cell lines by western blotting using polyclonal rabbit anti-human CD55 and anti-human CD59 antibodies (Santa Cruz Biotechnology) (Fig. S2). The parental and stably transfected SVAREC cells were maintained in DMEM with 10% FBS and 100 µg/mL hygromycin B.

### EV neutralization in the presence of complement

Serum from vaccinated mice or rabbit pAb or mAb was serially diluted 1∶3 in triplicate in serum free MEM in a round-bottom 96-well plate. Serum free MEM was added to all wells so that each well had 50 µL total volume. Next, ∼5×10^4^ PFU of EV containing a 1∶500 final dilution of anti-MV mAb 2D5 in MEM was added to each well (22 µL/well) so that each well had a total volume of 72 µL. Baby rabbit complement (C') (Cedarlane Laboratories, Burlington, NC) at a final dilution of 10% (8 µL) was added to each well so that the final volume in each well was 80 µL. Alternatively, heat inactivated baby rabbit C' (iC') was added as a negative control. The 96-well plates containing virus, antibody, and C' were incubated at 37°C for 30 minutes after which the contents from each well was transferred to Costar 96-well white clear-bottom tissue culture treated plates (Corning) containing a monolayer of BSC-1 cells. The BSC-1 cells were prepared on these plates 48 hours before use and subsequently used at 100% confluency. Once infected, the plates were incubated at 37°C for ∼20 hours. Luciferase production was measured by adding 100 µL of SuperLight™ Luciferase Reporter Gene Assay Reagent (BioAssay Systems, Hayward, Ca) directly to each well and relative light unit (RLU) measurements obtained on a MLX Revelation microtiter plate luminometer (Dynex Technologies). To relate RLU readings to PFU, known amounts of EV were serially diluted on the same BSC-1 plate to generate a standard curve. Linear fit was calculated and RLU readings were converted to PFU.

Neutralization of EV with human C' (Sigma-Aldrich, St. Louis, MO) or human C' depleted of C1q (Complement Technology, Tyler, TX) or C5 (Sigma-Aldrich, St. Louis, MO) was performed using the same luciferase assay as above with the following modifications. Prior to the addition of C', virus and antibody were incubated for 1 hour. After the addition of human C' (used at a concentration of 20%), the plate was incubated for an additional 1 hr. These changes were made because we found that neutralization using human C' with the rabbit and mouse antibodies was less efficient than with baby rabbit C'. Percent neutralization was calculated by dividing luciferase RLU readings from wells containing antibody by RLU readings of control wells containing no antibody for each serially diluted antibody. Percent neutralization by virolysis was calculated by the following formula: 100 – [(%NAb – %NAb with C5defC')/(%NAb – %NAb with hC')*100] where %NAb is percent neutralization with antibody, C5defC' is C5 deficient human C' and hC' is complete human C'. This formula controls for any neutralization with antibody alone and determines the contribution of the lytic pathway.

### Statistics

Statistical significance was determined using Prism 5.0 software. Differences in percent neutralization and weight loss were calculated using an unpaired 2-tailed t-test. Differences in survival were calculated using Kaplan-Meier analysis and log-rank test. *P* values of less than 0.05 were considered significant.

## Results

### Protection from lethal VACV challenge requires the inclusion of CpG adjuvant and correlates with the induction of IgG2a antibody

We have previously shown that vaccinating mice and non-human primates with a combination of VACV proteins and CpG and aluminum hydroxide (alum) protects from a lethal poxvirus infection [32], [34]. Using different adjuvant systems (MPL+TDM or QS21), Fogg et al. showed that vaccination with combinations of proteins provided better protection than individual proteins [23]. Here, we investigated the protection of BALB/c mice vaccinated with both combination and individual proteins with adjuvants CpG/alum or alum alone (Table 1). Mice that received a vaccine adjuvanted only with alum succumbed to infection, regardless of the combination of proteins given. Mice receiving protein(s) with CpG/alum were able to survive infection to varying degrees, with combination proteins achieving 100 percent survival. The combination protein vaccines achieved 100 percent protection due to antibody generated against both MV and EV. L1/CpG/alum and B5/CpG/alum showed partial protection. Notably, while A33/CpG/alum showed no survival at this challenge dose, the addition of A33 to L1/CpG/alum resulted in less post-challenge weight loss than mice vaccinated with only L1/CpG/alum (2% vs. 23% weight loss; p = 0.0003) again demonstrating that combinations of proteins provided better protection than individual proteins.

**Table 1:** Summary of survival and maximum weight loss after challenge with 4×10^6^ pfu VACV.

Vaccination group^a^	Percent survival	Maximum percent average weight loss^b^
ABL/CpG/alum	100	10
AL/CpG/alum	100	2
BL/CpG/alum	100	12
L1/CpG/alum	80	23
B5/CpG/alum	40	24
A33/CpG/alum	0	N/A
ABL/alum	0	N/A
AL/alum	0	N/A
BL/alum	0	N/A
L1/alum	0	N/A
B5/alum	0	N/A
A33/alum	0	N/A
unvaccinated	0	N/A

a. A, B, L: A33, B5, L1, respectively. Alum: aluminum hydroxide.

b. N/A: not applicable. Since these groups had 0% survival, average weight loss of the group is not reported since mice were sacrificed when they had 30% weight loss or died prior to reaching this degree of weight loss.

**Figure 1:**
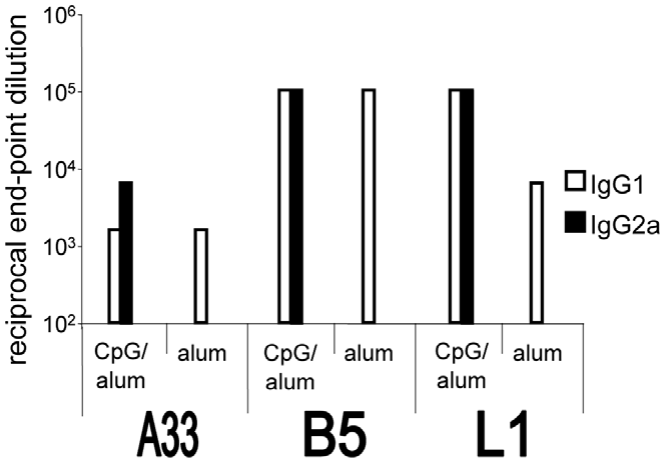
Antibody isotypes produced in BALB/c mice after vaccination. Groups of 6-week old female BALB/c mice (9 mice/group) vaccinated individually with A33, B5, or L1 adjuvanted with alum +/− CpG were bled three weeks after the boost vaccination. Equal volumes of heat-inactivated serum from individual mice in each group were pooled. Shown are reciprocal end-point dilutions for antibody isotypes IgG1 (white bars) and IgG2a (black bars) as measured by ELISA reactivity with proteins A33, B5, or L1. Vaccinations without CpG produced no detectable IgG2a response.

Others have previously shown that the addition of CpG adjuvant biases the immune response towards Th1 in mice [33], [51]–[54]. As expected, IgG2a antibody was only produced in BALB/c mice given the CpG/alum combination adjuvant (Fig. 1). IgG1 was produced in both CpG/alum and alum only groups, with varying titers to the individual proteins.

### Sera from mice vaccinated with A33 or B5 /CpG/alum can neutralize large numbers of EV particles in the presence of complement

**Figure 2:**
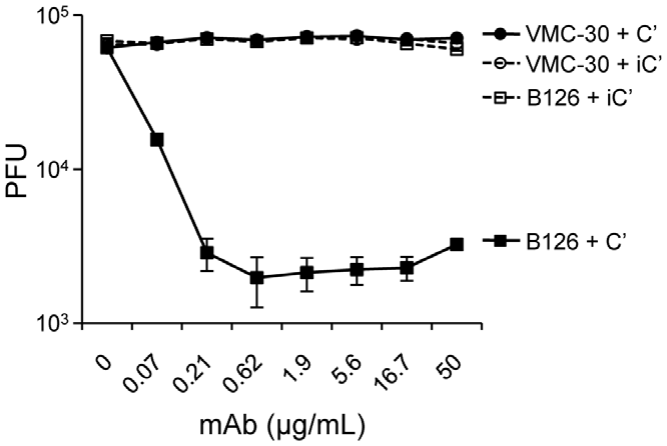
Neutralization of large numbers of EV particles using anti-B5 mAbs and complement. Anti-B5 mAb B126 (squares) and anti-B5 mAb VMC-30 (circles) were used in a luciferase-based high PFU EV neutralization assay. ∼5×10^4^ pfu of EV was incubated with increasing concentrations of mAb in the presence of 10% baby rabbit C' (solid symbols) or heat inactivated baby rabbit C' (iC') (open symbols). Luciferase units were converted to PFU by linear regression of a standard curve using known numbers of EV. Neutralization was performed in triplicate for C' and duplicate for iC' and represents two independent experiments. Error bars represent standard deviation.

Given the correlation seen between the appearance of Th1-biased antibodies (IgG2a in mice) and protection, as well as previous studies showing that Th1-biased antibodies are more protective than Th2-biased antibodies and can neutralize EV in the presence of C' [33], [34], [42], [43], we next determined if sera from vaccinated mice could neutralize EV in the presence of C'. Previously, plaque reduction assays with small numbers of EV (∼50–150 PFU) were used to show neutralization; however, we wished to test the ability of antibody and C' to neutralize large numbers of EV particles that are more likely present during an infection. To do this, we developed an EV neutralization assay with a recombinant VACV that expressed a luciferase reporter protein. For this assay we generated standard curves with known amounts of EV, which allowed for conversion of RLU to PFU. In this assay, anti-L1 mAb 2D5 [48] was always added to neutralize contaminating MV in the EV preparation. To confirm the functionality of this assay, we tested B126, an anti-B5 mAb with IgG2a isotype previously shown to neutralize EV in the presence of C' [43], as well as VMC-30, an anti-B5 mAb from a previously characterized panel of anti-B5 mAbs [55]. As shown in Fig. 2, B126 was capable of neutralizing 97% of >10^4^ EV particles in the presence of C'; however, VMC-30 was not. Neutralization was abrogated if C' was first heat inactivated.

**Figure 3:**
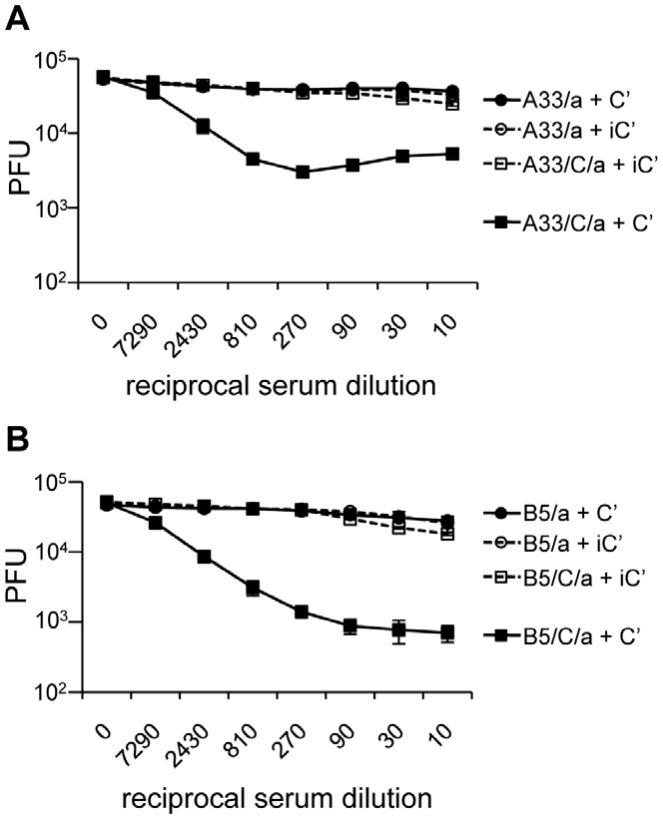
Neutralization of large numbers of EV particles using sera from mice vaccinated with A33 or B5 in CpG/alum or alum only. Sera from BALB/c mice vaccinated with A33 (A) or B5 (B) adjuvanted with CpG/alum (squares) or alum only (circles) were collected 3 weeks post boost vaccination. Equal volumes of heat-inactivated serum from groups of mice were pooled (9 mice/group). ∼5×10^4^ pfu of EV was neutralized with increasing amounts of sera in the presence of 10% baby rabbit C' (closed symbols) or heat inactivated baby rabbit C' (iC') (open symbols). Luciferase units were converted to PFU by linear regression of a standard curve using known numbers of EV. Neutralization was performed in triplicate for C' and duplicate for iC' and represents three independent experiments. Error bars represent standard deviation.

Using this luciferase-based high EV particle neutralization assay, we found that anti-A33 and anti-B5 sera from mice vaccinated with protein and CpG/alum could neutralize large numbers of EV particles in the presence of C' (Fig. 3). Neutralization was largely abrogated if C' was first heat inactivated. Anti-B5 sera was better at C'-mediated neutralization than anti-A33 sera, though this might be explained by the higher titer of IgG2a anti-B5 antibody compared to anti-A33 antibody (Fig. 1). Interestingly, while anti-A33 antibodies have the ability to neutralize EV in the presence of C', there was a lack of protection in mice vaccinated with A33/CpG/alum.

### Anti-A33 C'-mediated antibody neutralization requires steps that could lead to EV outer envelope lysis, while anti-B5 C'-mediated antibody neutralization can occur by opsonization

**Figure 4:**
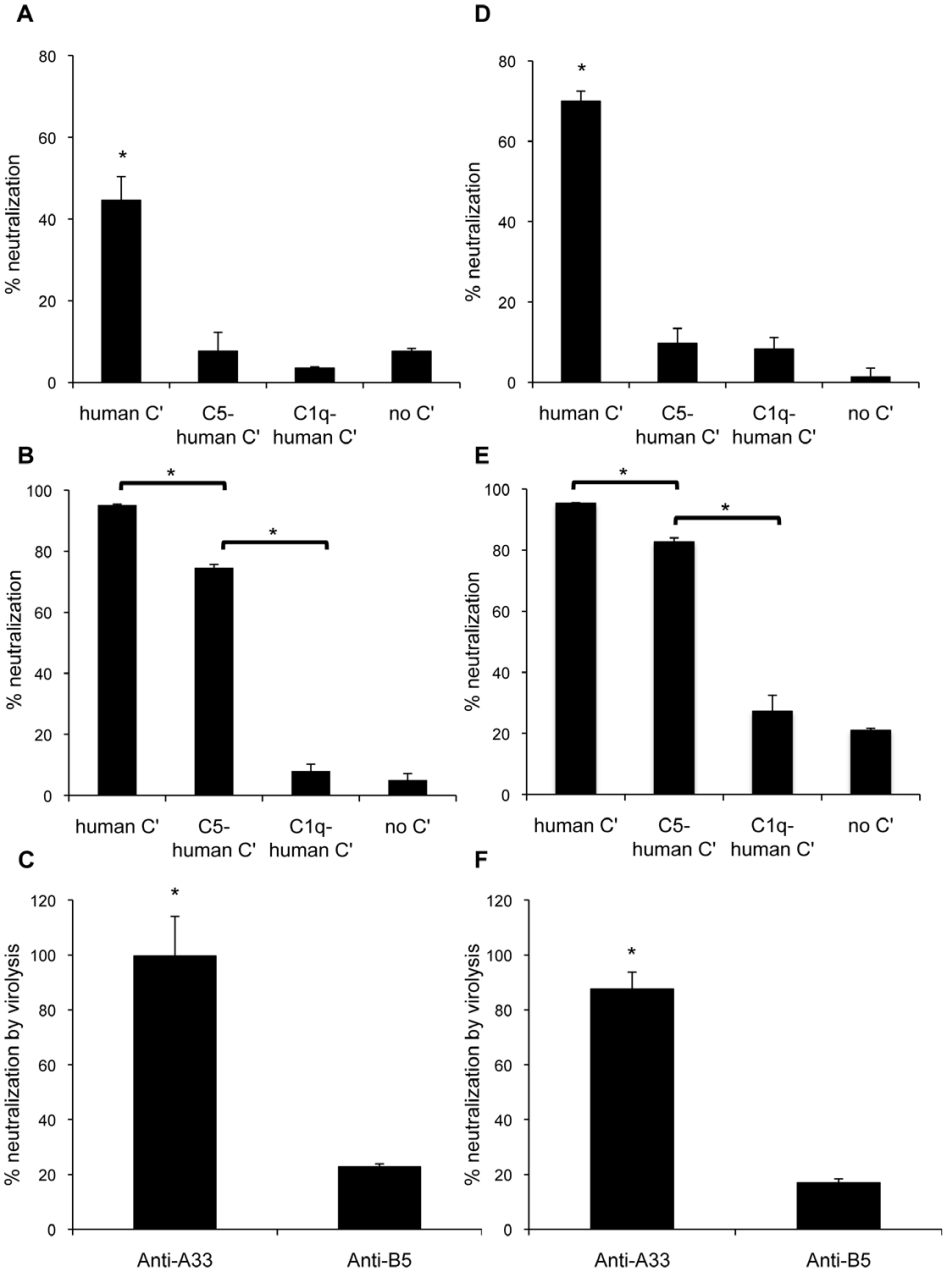
Contribution of steps leading to the formation of the membrane attack complex in complement-mediated neutralization of EV. Graphed is the percent neutralization of EV in the presence of antibody and 20% human C' or with human C' depleted of component C5 or C1q. Sera from BALB/c mice vaccinated with A33 or B5/CpG/alum was used at a dilution of 1∶20 (A–C). Rabbit pAb against A33 and B5 was used at 50 µg/mL (D–F). Data shown in A, B, D, and E are percent neutralization of no antibody control. Data in C and F show the specific EV neutralization dependent on C5 (virolysis) and was calculated as described in materials and methods. Neutralizations were performed in triplicate. Error bars represent standard deviation. * p<0.05.

To elucidate why the A33/CpG/alum vaccination was not as effective as B5/CpG/alum at protecting mice from lethal challenge despite neutralizing EV in the presence of C', we asked whether the pathway of C'-mediated neutralization was playing a role. Lustig, et al. demonstrated that anti-A33 polyclonal rabbit sera (pAb) resulted in C'-mediated lysis of the outer membrane of EV allowing anti-MV neutralizing antibody access to the MV particle within [31]. Benhnia et al. showed that anti-B5 mouse mAb B126 neutralized EV by opsonizing particles with complement and could neutralize in the absence of membrane attack complex formation and without an anti-MV antibody present [43]. However, the question remained whether these observations with anti-A33 and anti-B5 antibodies were due to the differing EV target proteins or if the differences in C'-mediated neutralization was due to inherent differences in the two studies (e.g., pAb vs. mAb, rabbit vs. mouse antibodies, and different adjuvants used during generation of the antibodies). To determine if different C'-mediated neutralization pathways might be used for different EV protein targets, sera from mice vaccinated with either A33 or B5 /CpG/alum were used with human C' depleted for C5 or C1q (Figs. 4A–C). Depletion of either C1q or C5 from sera significantly reduced the ability of anti-A33 mouse sera to efficiently neutralize EV (Fig. 4A) indicating that steps that lead to formation of the membrane attack complex were required for neutralization. However, only the sera depleted of C1q affected the ability of anti-B5 mouse sera to neutralize EV (Fig. 4B) indicating that membrane attack complex does not need to form for successful EV neutralization. Similar results were obtained when rabbit pAb against A33 and B5 was used (Figs. 4D and E). These results are consistent with the previously described findings that the mechanism of C'-mediated neutralization for A33 and B5 differ[31], [43], with anti-A33 antibody relying on virolysis and anti-B5 antibody able to neutralize by opsonization (Figs. 4C and F).

**Figure 5:**
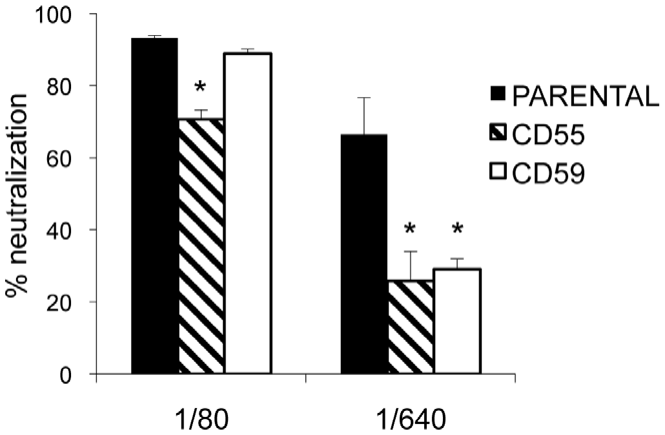
Protection of EV by human regulators of complement from complement-mediated neutralization at high and low amounts of antibody. Sera from BALB/c mice vaccinated with B5/CpG/alum was used to neutralize EV produced in SVAREC cells expressing CD55 (striped bars), CD59 (white bars), or no human regulators of C' (black bars) in the presence of 20% human C'. While a full range of antibody dilutions were tested, shown is a representative low dilution (1/80) and high dilution (1/640) of antibody and the effect on complement-mediated neutralization of EV containing CD55 or CD59. The full range of antibody dilutions revealed that EV was partially protected by CD59 at dilutions between 1/80 and 1/640, but not as protective as CD55 at those dilutions. Data is shown as percent neutralization of no antibody control. Error bars represent standard deviation. * p<0.05.

Benhnia hypothesized that antibody alone was unable to fulfill the basic occupancy model for EV neutralization because of the amount of B5 protein on the EV surface and that antibody-induced C' coating of the EV membrane allowed for the occupancy model to succeed [42]. To examine this further, we varied the anti-B5 antibody concentration as well as used EV that incorporated different C' regulators into its outer envelope. These C' regulators (CD55 and CD59) have been shown to be present on the EV envelope [49]. CD55, also known as decay-accelerating factor, inhibits stable formation of the C3 convertase and down-modulates the amount of C3b/C4b deposition as well as the downstream steps in the C' cascade [56] and thus could alter the ability of C' to opsonize EV. CD59, or protectin, prevents formation of the membrane attack complex and could block virolysis of the EV membrane. We produced EV that had each C' regulator on its surface using previously described SVAREC cell lines that expressed no human C' regulators or were stably transfected to express human CD55 or CD59[49], [50]. At high concentrations of anti-B5 sera (1∶80), EV produced in the cell line expressing CD55 showed some protection from C'-mediated neutralization (Fig. 5, striped bar), while EV produced in the cell line expressing CD59 showed no protection from C'-mediated neutralization (Fig. 5, white bar). This data supports a model where virolysis is not needed since the presence of CD59 did not alter the ability to neutralize EV at the relatively high concentration of antibody. However, at relatively low concentrations of anti-B5 sera (1∶640), EV produced in the cell lines expressing CD55 or CD59 were equally protected from C'-mediated neutralization (striped and white bars). The finding that CD59 provides protection equal to that of CD55 when antibody is at low concentration indicates that virolysis becomes the predominant pathway for neutralization. Therefore, if the amount of antibody on the surface of EV is limited, virolysis is required for neutralization as was seen with anti-A33 sera.

### Complement is partially responsible for the protection seen in mice after passive or active immunization

**Figure 6:**
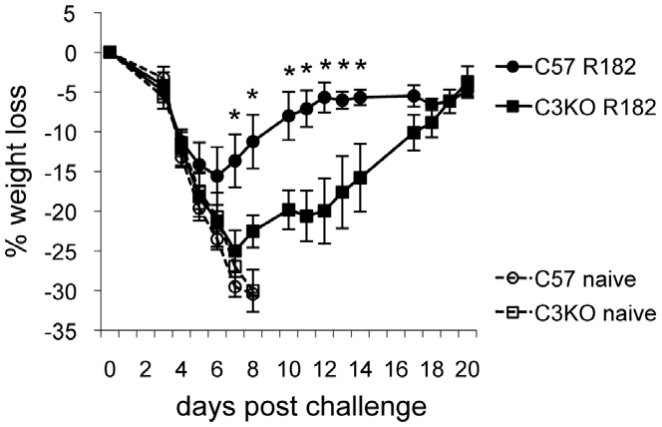
Protection from vaccinia virus challenge by anti-B5 rabbit pAb in C57BL/6 and C3KO mice. Anti-B5 rabbit pAb (R182; 2 mg/mouse) were passively transferred by the i.p. route into 9-week old female C57BL/6 (circles) and C3KO (squares) mice. Groups of mice that did not receive antibody treatment were included (dashed lines and open symbols). Twenty-four hours after antibody treatment, mice were i.n. challenged with ∼9×10^4^ pfu of vaccinia virus. Weight loss was monitored and the percent weight loss calculated against each mouse's starting weight. Five of 5 mice in each R182 treated group survived challenge while 4 of 4 naïve C57BL/6 and 3 of 4 naïve C3KO mice did not. Error bars represent standard error. * p<0.05.

**Figure 7:**
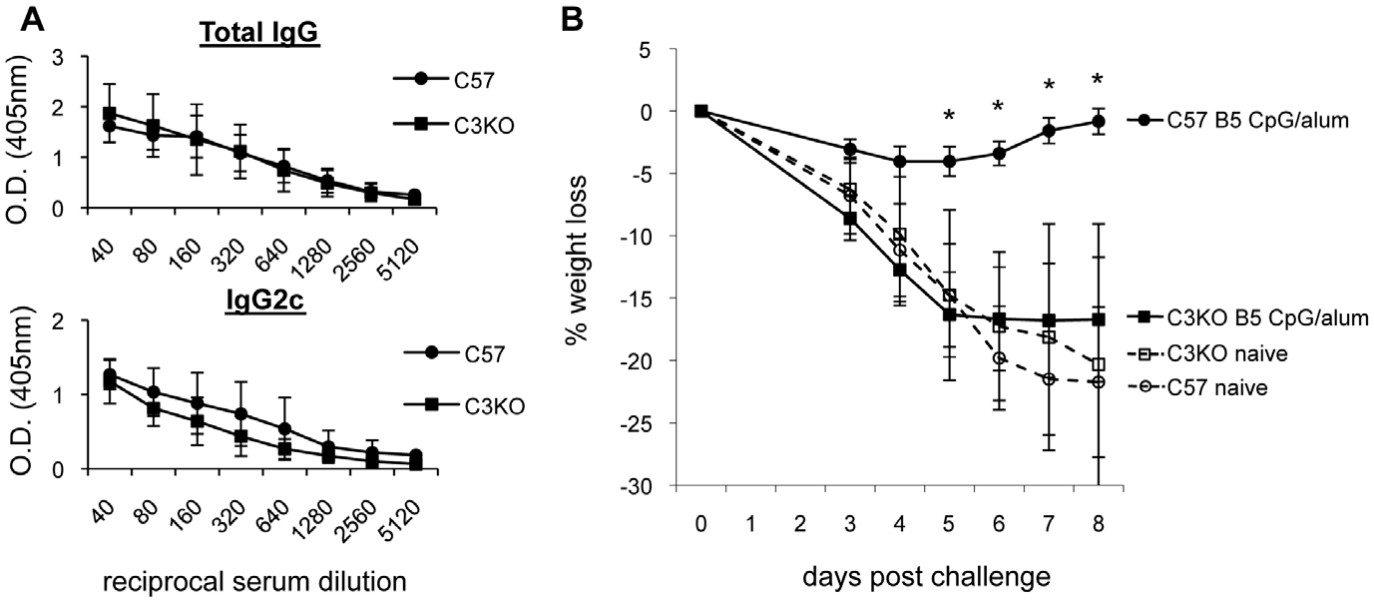
Protection from vaccinia virus challenge by B5/CpG/alum vaccination in C57BL/6 and C3KO mice. 9-week old male C57BL/6 (circles) (5/group) and C3KO (squares) (6/group) mice were vaccinated with B5/CpG/alum. (A) Anti-B5 total IgG and IgG2c were measured by ELISA from sera taken from vaccinated mice 3 weeks after boost vaccination. Because C57BL/6 mice do not have the gene for IgG2a, IgG2c was measured and is known to have similar effector functions [58], [74], [75]. (B) Three weeks after the boost vaccination, mice were i.n. challenged with ∼2×10^5^ pfu of vaccinia virus. Unvaccinated naïve C57BL/6 and C3KO groups were included (3 mice/group) (dashed lines and open symbols). Weight loss was monitored and the percent weight loss calculated against each mouse's starting weight. One of the B5/CpG/alum vaccinated C57BL/6 mice lost significantly more weight than the other 4 mice in its group and was removed from analysis based on Grubbs' test for outlier detection. Error bars represent standard error. Data shown is representative of two independent experiments. * p<0.05. At time of challenge, the mice were ∼14 weeks old and at this challenge dose in the C57BL/6 background only about half of the unvaccinated mice required euthanasia. Thus, mortality between groups was not statistically significant.

To determine what effector functions of antibody were important for in vivo protection, we used immunizations that targeted B5. Benhnia et al. found that passive immunization with mAb B126 was less protective in vivo if mice were first transiently depleted of C' using cobra venom factor (CVF) [43]. However, mice still recovered and were more protected than naïve mice. To first confirm the contribution of C' in protection by a polyclonal anti-B5 antibody response, we passively transferred pAb rabbit anti-B5 antibody (R182) into C3 knockout (C3KO) and wild-type mice (C57BL/6) and intranasally (i.n.) challenged them with a lethal dose of VACV (Fig. 6). C3KO mice lost significantly more weight than C57BL/6 mice indicating that C' contributed to protection. However, similar to Benhnia et al. [43], when compared to the controls that were not treated with antibody, we did note partial protection in the absence of C'. Next, to determine if antibodies produced during active immunization protected in a similar fashion, we vaccinated C3KO and wild-type C57BL/6 mice with B5/CpG/alum. Interestingly, despite a few reports that C3KO mice were defective in making antibody responses [44], [57]–[59], we found that our vaccine resulted in total IgG and IgG2c responses comparable to the wild type C57BL/6 mice (Fig. 7A). After vaccinations, mice were challenged i.n. with VACV and weight loss was monitored. Vaccinated C57BL/6 mice lost minimal weight and fully recovered by day 8 post-infection. Conversely, vaccinated C3KO mice lost significant weight similar to C57BL/6 naïve and C3KO naïve mice (Fig. 7B) again indicating a role of C' in protection from challenge.

### Fc receptors protect mice passively transferred with anti-B5 antibody in the absence of complement

**Figure 8:**
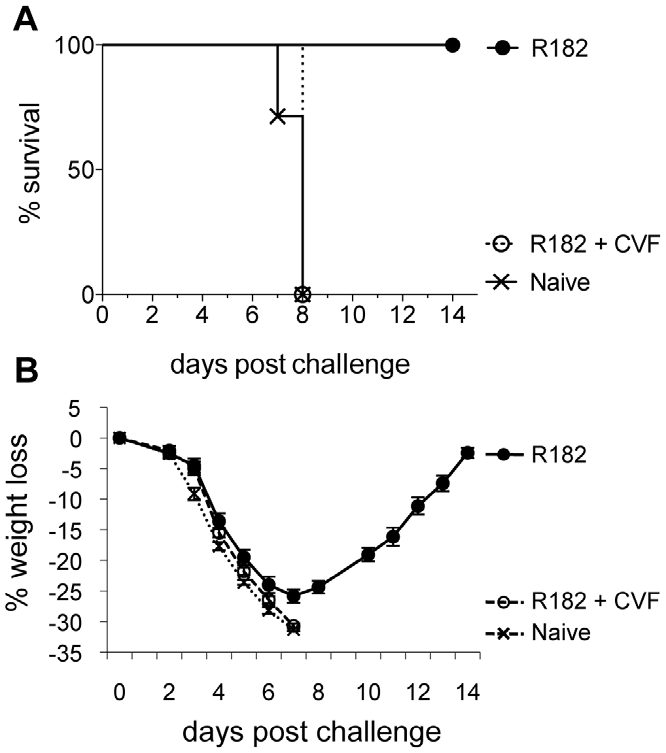
Protection from vaccinia virus challenge by anti-B5 rabbit pAb in FcRKO mice. Anti-B5 rabbit pAb (R182; 2 mg/mouse) were passively transferred by the i.p. route into 7- to 13-week old female FcRKO mice (8 mice/group; circles). Approximately 4 units of cobra venom factor were delivered on days −1, 2, and 5 by the i.p. route to one group of mice that received R182 (open circles). A group FcRKO mice that did not receive antibody or CVF was included (crosses). Twenty-four hours after antibody treatment, mice were i.n. challenged with ∼3×10^5^ pfu of vaccinia virus. (A) Survival differences between the FcRKO mice treated with R182 and those treated with R182 and CVF were statistically significant; p = 0.0084 (Log-rank Test). (B) Weight loss was monitored and percent weight loss calculated against each mouse's starting weight. Error bars represent standard error. Data shown is representative of two independent experiments.

**Figure 9:**
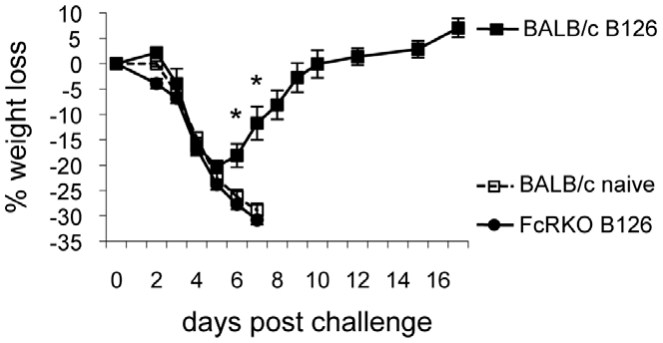
Protection from vaccinia virus challenge by anti-B5 mAb B126 is FcR dependent. Anti-B5 mAb (B126; 100 µg) was passively transferred by the i.p. route into 8- to 9-week old female FcRKO (closed circle) and 8-week old female BALB/c mice (closed squares) (4 mice/group). A naïve female BALB/c control group was included (dashed line, open square). Twenty-four hours after antibody treatment, mice were i.n. challenged with ∼4×10^5^ pfu of vaccinia virus. Weight loss was monitored and percent weight loss calculated against each mouse's starting weight. Error bars represent standard error. Data shown is representative of two independent experiments. * p<0.05. All BALB/c mice given B126 survived challenge, while FcRKO mice given B126 and untreated BALB/c mice all succumbed to infection and/or reached end-point criteria for euthanasia.

In the work by Benhnia, et al [43], it was unclear whether the recovery of normal levels of C' after CVF depletion or effector functions mediated through Fc receptors (FcRs) were responsible for the protection afforded by mAb B126 even after C' depletion. Based on our findings in C3KO mice, it was evident that additional mechanisms were playing a role in protecting mice in the absence of C'. Thus, we passively transferred FcRKO mice with anti-B5 pAb (R182) and i.n. challenged them with a lethal dose of VACV (Fig. 8). These mice lost significant weight (∼25%) but all survived challenge. However, if FcRKO mice were transiently depleted of C' with CVF before challenge, all mice succumbed to infection indicating that both FcR and C' play a role in protection (Fig. 8B). Given the finding that FcRs play a role in protection with rabbit polyclonal anti-B5 antibody, we sought to determine if mAb B126 also used FcRs to protect mice from lethal challenge. B126 was passively transferred into FcRKO or wild type BALB/c mice followed by i.n. challenge with a lethal dose of VACV (Fig. 9). By day 6, significant differences were seen in the weight loss of these BALB/c and FcRKO mice treated with B126. By day 8, B126 treated FcRKO mice had succumbed to infection while the wild type BALB/c mice that received B126 had already started to recover and ultimately survived challenge again indicating an important role for FcR in protection after passive immunization.

## Discussion

Vaccine induced antibodies have been shown to be critical for protection from orthopoxvirus challenge [14], [60]–[62]. Likewise, protection afforded by protein vaccination is thought to heavily depend on antibody responses generated and often these responses are measured and reported as a possible correlate of protection [15], [23], [28], [30], [32]–[34]. However, besides direct pathogen neutralization, these antibody responses could protect through various effector mechanisms such as activation of C' to neutralize virus or lyse infected cells and activating cellular responses through FcRs to lyse and kill infected cells. Here, we examined the functionality of antibody responses generated after vaccination with individual VACV proteins to better understand the type of antibody response needed to confer protection. Recently, Benhnia et al. showed that a mouse anti-B5 mAb named B126 required C' to neutralize VACV EV and mediate in vivo protection [43]. Therefore, we examined whether the same was true for pAb responses after active or passive immunization against the VACV EV protein B5.

To do this, we initially set up a new luciferase based assay to measure the neutralization of large numbers of EV particles. B126 was confirmed to neutralize EV in the presence of C' (Fig. 2) as had been previously reported [43]. We also tested another anti-B5 mAb (VMC-30; [55]) and found that it was unable to neutralize EV in the presence of C' (Fig. 2). Benhenia et al, reported that B126 was an IgG2a isotype and this afforded it the ability to activate C' as mAbs of IgG1 isotype did not [43]. Interestingly, VMC-30 is an IgG2b isotype [55], which should also be capable of activating C' and other effector functions similar to IgG2a [63]–[66]. However, in this case, the isotype of the mAb did not predict functional activity in vitro and therefore highlights the importance of testing functional activity of mAb and not relying solely on the prediction of isotype analysis. When passively transferred into mice, VMC-30 also did not protect against challenge (data not shown), again demonstrating the need for effector function for protection in vivo. We confirmed the isotype of VMC-30 and speculate that it may have been unable to neutralize VACV in the presence of C' due to potential amino acid changes in the Fc region of the mAb which could abrogate functional activity [67], [68]. However the IgG2b isotype could interact with Fc receptors, so other factors like affinity may be playing a role in its inability to protect. The role of IgG2b in mediating protection from vaccinia virus infections in vivo is currently unknown. This may be interesting to examine further in the future.

As others had previously found in BALB/c mice [33], we observed that protection in vivo by protein vaccination was correlated with the production of IgG2a antibodies (Table 1 and Fig. 1). Therefore, we predicted that these antibodies would neutralize EV in the presence of C' similar to B126, but we needed to fully examine this given the lack of C'-fixing activity with mAb VMC-30. We found that sera containing IgG2a from mice vaccinated with A33 or B5/CpG/alum could utilize C' to neutralize large numbers of EV particles in vitro. Sera lacking IgG2a antibody from mice vaccinated with proteins and alum only was unable to neutralize virus in the presence of C' (Fig. 3). This confirms the importance of isotype and strengthens the correlation between protection of mice and production of IgG2a isotype antibodies.

Further examination of the mechanism of C'-mediated neutralization revealed that anti-A33 and anti-B5 antibody responses utilized C' to neutralize EV in different ways (Fig. 4). In agreement with previous reports [31], [42], [43], A33 antibody required C' activation steps that could lead to virolysis, while B5 antibody and C' could neutralize through opsonization. Benhnia et al. provided a model of anti-B5 antibody- C' mediated neutralization whereby B5 protein was not in high enough density on the surface of EV to allow for the basic occupancy model of antibody neutralization to succeed [42], [43]. They reasoned that deposited C' components enhanced the footprint of antibody bound to B5 protein on the virus surface to assist in neutralization. This model explained why virolysis was not needed. Lustig et al. provided a model of C' assisted EV neutralization for anti-A33 antibody [31]. In this model, C' lyses the outer envelope of EV, which provides anti-MV antibody access to the MV virion within. An anti-MV antibody was required to be present during the assay for C' assisted neutralization to occur. Because our luciferase based EV neutralization assay always contains an anti-MV antibody to eliminate contaminating MV in the EV preparations, we were unable to examine neutralization in the absence of an anti-MV antibody. Despite this, our data suggests that both mechanisms are correct for each protein target and not due to differences in antibody species, clonality, or adjuvant used to generate the antibody.

We hypothesized that disparity in A33 and B5 protein density on the EV surface contributed to the difference in mechanism. Galmiche et al. showed that total EV lysate had A33 and B5 protein amounts of <5 µg/mg and 30 µg/mg, respectively [15]. The reduced amount of A33 protein on the EV surface could decrease the amount of antibody bound to EV to the point where coating with C1q and C3b/C4b in the area around the bound antibody is still insufficient to completely opsonize the EV virion. Under this scenario, formation of even one or two membrane attack complexes (MAC) on the EV virion could be enough to disrupt the outer membrane and allow access of neutralizing MV antibody. This model would predict that further limiting the amount of anti-B5 antibody bound to the EV surface (equivalent to a lower density of protein) would switch the mechanism of C'-mediated neutralization from opsonization to lysis. To test this hypothesis, we used a novel approach whereby EV was generated with the incorporation of different human regulators of C' (Fig. 5). We found that when EV was generated in cells that would result in the inclusion of CD59 (an inhibitor of MAC formation) on EV, CD59 could not provide additional protection from C'-mediated neutralization at high concentrations of anti-B5 antibody as neutralization could occur through opsonization. However, at low concentrations of anti-B5 antibody, CD59 was protective against C'-mediated neutralization to the same degree as EV containing CD55 (an inhibitor of C3 convertase formation), likely indicating the mechanistic switch from opsonization to lysis. Additionally, we found that under the right experimental conditions, human regulators of C' on the VACV EV surface can block C' activation by antibody, and not just activation by C' alone [49]. These findings provide new insight into interactions of antibody, C', and viral protein and how those interactions impact neutralization of virus.

The finding that A33 requires virolysis for C'-mediated neutralization while B5 does not may also explain differences in protection we observed after vaccinating with A33 or B5/CpG/alum. At the challenge doses we used, the ability of B5 to provide at least partial protection could be explained by the ability to neutralize EV in the absence of an anti-MV antibody response, which A33 is incapable. A33 antibody and C' would simply release MV particles which could propagate the infection, albeit that some anti-A33 effect could be gained by allowing C' free access to the C' sensitive MV particle or A33 antibody-dependent lysis of infected cells. This may also explain why a vaccine that adds L1 to A33 improves protection from disease compared to A33 or L1 alone (Table 1).

To examine more closely which effector functions of antibodies are important for protection in vivo, we studied the role of C' and FcRs in the protection we observed with B5 antibody. The rabbit anti-B5 pAb used in neutralization experiments had been previously shown to be protective in vivo by passive immunization [26] and the ability to neutralize EV in the presence of C' potentially contributed to this observation. To confirm this, we examined the ability of anti-B5 antibody to protect mice in the absence of the central C' component C3 (Figs. 6 and 7). We found that both passive immunization with rabbit anti-B5 antibody and active immunization with B5/CpG/alum partially relied on C' for protection. Similar to previously reported studies that transiently depleted C' in challenged animals [43], [69], we found that antibody could still provide partial protection even in the genetic absence of C3, which abrogates the function of the C' system (Figs. 6 and 7B). Somewhat unexpectedly, we found that vaccinated C3KO mice generated antibody responses similar to that of wild-type mice (Fig. 7A). Binding of the B cell antigen receptor/co-receptor by C3d-antigen complexes lowers the threshold for B cell activation by 10- to 100-fold [70] and provides an important survival signal to B cells [71]. CpG has been shown to directly stimulate B cells and enhance IgG secretion [72], [73]. Inclusion of CpG in our vaccine may stimulate B-cells in a way that overcomes the requirement of C' activation for B-cell priming, activation, and survival.

Because we observed partial protection in the absence of C', we examined whether FcRs may be playing a role in protection as was previously suggested by Benhnia et al. [43]. FcRKO mice were partially protected by passive transfer of rabbit anti-B5 pAB, but not if C' was transiently depleted with CVF first (Fig. 8). Likewise, anti-B5 mAb B126 was heavily reliant on FcRs for its protective effects (Fig. 9). This finding indicates that both C' and FcRs can contribute to protection and that both are important effector functions that mediate protection by pAb anti-B5 responses in vivo.

In summary, we found that after active vaccination, pAb responses against the EV form of VACV utilize C' and FcRs to mediate protection. C' plays an important role in neutralization and the protein target can alter the mechanism through which this neutralization occurs. FcRs contribute to protection in vivo likely through Fc mediated phagocytosis and/or ADCC. Together these effector functions cooperate to provide protection from challenge. Importantly, we suggest the need to evaluate antibody effector function requirements for protection in vivo to any pathogen, especially if monoclonal antibodies are to be used. Advances in the understanding of the molecular basis for effector functions of antibody allows for customization. By altering the Fc region amino acid sequence one can impart or abrogate specific effector functions [67]. By understanding the mechanism by which antibodies provide protection against a given pathogen and understanding how to manipulate antibody effector functions, vaccines and other therapeutic antibodies can be designed to specifications that activate C' or FcRs as necessary.

## Supporting Information

Figure S1 **Cobra Venom Factor (CVF) treatment of BALB/c mice transiently depletes complement.** To assess the degree of C' depletion after CVF treatment, groups of 11- to 12-week old female BALB/c mice (2 mice per group) were treated with CVF and then terminally bled the following day. One group was treated on day -1 and bled the next day (d0). A second group was treated on days -1 and +2 and bled the next day (d3), A third group was treated on days −1, +2, and +5 and bled the next day (d6). A group of untreated mice was used a control (No CVF). (A) CH50 assay using rabbit erythrocytes (Complement Technology, Tyler, TX) sensitized with goat anti-rabbit erythrocyte antibody (MP Biomedicals, Solon, OH) was performed with sera from mice treated or not treated with CVF. Complement activity levels on days 0 and 3 were low, while some complement activity returned by day 6. Note that sensitized rabbit erythrocytes were used because sensitized sheep erythrocytes are resistant to lysis by mouse complement. (B) Western blot of C3 protein in sera of mice treated or not treated with CVF. Serum (2 µl) from the indicated group of mice was loaded on to a 10% polyacrylamide gel. After blotting, HRP-conjugated goat anti-mouse C3 antibody (MP Biomedicals, Solon, OH) at 1∶10,000 was used to probe for the presence of C3. C3 protein was not detected on days 0 and 3, while some C3 protein was detected on day 6.
(TIF)

Figure S2 **CD55 and CD59 are detected in stably transfected SVAREC cell lines.** SVAREC cell lines stably transfected to express CD55 and CD59 [49], [50] were grown under selective pressure as described in materials and methods. Western blotting for either CD55 (αCD55) or CD59 (αCD55) was performed on lysates from the parental SVAREC cell line expressing no human complement regulators (Par), a SVAREC cell line expressing CD55 (CD55), and a SVAREC cell line expressing CD59 (CD59). Rabbit polyclonal anti-human CD55 and anti-human CD59 antibodies were used at a dilution of 1∶250. CD55 was only detected in the CD55-expressing cell line at its expected size of ∼70 kDa and CD59 was only detected in the CD59-expressing cell line at its expected size of ∼20 kDa.
(TIF)

## References

[1] Fenner F, Henderson D, Arita I, Jezek Z, Ladnyi I (1988). Smallpox and its eradication..

[2] Moore ZS, Seward JF, Lane JM (2006). Smallpox.. Lancet.

[3] Nalca A, Zumbrun EE (2010). ACAM2000: the new smallpox vaccine for United States Strategic National Stockpile.. Drug Des Devel Ther.

[4] Bradbury J (2001). USA to increase smallpox vaccine stockpile.. Lancet Infect Dis.

[5] Fulginiti VA, Papier A, Lane JM, Neff JM, Henderson DA (2003). Smallpox vaccination: a review, part II. Adverse events.. Clin Infect Dis.

[6] Poland GA, Grabenstein JD, Neff JM (2005). The US smallpox vaccination program: a review of a large modern era smallpox vaccination implementation program.. Vaccine.

[7] Beachkofsky TM, Carrizales SC, Bidinger JJ, Hrncir DE, Whittemore DE (2010). Adverse events following smallpox vaccination with ACAM2000 in a military population.. Arch Dermatol.

[8] Paran N, Sutter G (2009). Smallpox vaccines: New formulations and revised strategies for vaccination.. Hum Vaccin.

[9] Kennedy RB, Ovsyannikova I, Poland GA (2009). Smallpox vaccines for biodefense.. Vaccine.

[10] Cohen ME, Isaacs SN, Levine MM (2009). Improved Smallpox Vaccines in New Generation Vaccines;.

[11] Kennedy JS, Greenberg RN (2009). IMVAMUNE: modified vaccinia Ankara strain as an attenuated smallpox vaccine.. Expert Rev Vaccines.

[12] Riedmann EM (2010). FDA Fast Track status for IMVAMUNE.. Hum Vaccin 6:.

[13] Belyakov IM, Earl P, Dzutsev A, Kuznetsov VA, Lemon M (2003). Shared modes of protection against poxvirus infection by attenuated and conventional smallpox vaccine viruses.. Proc Natl Acad Sci U S A.

[14] Edghill-Smith Y, Golding H, Manischewitz J, King LR, Scott D (2005). Smallpox vaccine-induced antibodies are necessary and sufficient for protection against monkeypox virus.. Nat Med.

[15] Galmiche MC, Goenaga J, Wittek R, Rindisbacher L (1999). Neutralizing and protective antibodies directed against vaccinia virus envelope antigens.. Virology.

[16] Hammarlund E, Lewis MW, Hansen SG, Strelow LI, Nelson JA (2003). Duration of antiviral immunity after smallpox vaccination.. Nat Med.

[17] Amanna IJ, Slifka MK, Crotty S (2006). Immunity and immunological memory following smallpox vaccination.. Immunol Rev.

[18] Condit RC, Moussatche N, Traktman P (2006). In a nutshell: structure and assembly of the vaccinia virion.. Adv Virus Res.

[19] Payne LG (1980). Significance of extracellular enveloped virus in the in vitro and in vivo dissemination of vaccinia.. J Gen Virol.

[20] Payne LG, Kristensson K (1985). Extracellular release of enveloped vaccinia virus from mouse nasal epithelial cells in vivo.. J Gen Virol 66 ( Pt.

[21] Smith GL, Vanderplasschen A, Law M (2002). The formation and function of extracellular enveloped vaccinia virus.. J Gen Virol.

[22] Roberts KL, Smith GL (2008). Vaccinia virus morphogenesis and dissemination.. Trends Microbiol.

[23] Fogg C, Lustig S, Whitbeck JC, Eisenberg RJ, Cohen GH (2004). Protective immunity to vaccinia virus induced by vaccination with multiple recombinant outer membrane proteins of intracellular and extracellular virions.. J Virol.

[24] Hooper JW, Custer DM, Schmaljohn CS, Schmaljohn AL (2000). DNA vaccination with vaccinia virus L1R and A33R genes protects mice against a lethal poxvirus challenge.. Virology.

[25] Hooper JW, Thompson E, Wilhelmsen C, Zimmerman M, Ichou MA (2004). Smallpox DNA vaccine protects nonhuman primates against lethal monkeypox.. J Virol.

[26] Lustig S, Fogg C, Whitbeck JC, Eisenberg RJ, Cohen GH (2005). Combinations of polyclonal or monoclonal antibodies to proteins of the outer membranes of the two infectious forms of vaccinia virus protect mice against a lethal respiratory challenge.. J Virol.

[27] Wolffe EJ, Vijaya S, Moss B (1995). A myristylated membrane protein encoded by the vaccinia virus L1R open reading frame is the target of potent neutralizing monoclonal antibodies.. Virology.

[28] Fang M, Cheng H, Dai Z, Bu Z, Sigal LJ (2006). Immunization with a single extracellular enveloped virus protein produced in bacteria provides partial protection from a lethal orthopoxvirus infection in a natural host.. Virology.

[29] Chen Z, Earl P, Americo J, Damon I, Smith SK (2006). Chimpanzee/human mAbs to vaccinia virus B5 protein neutralize vaccinia and smallpox viruses and protect mice against vaccinia virus.. Proc Natl Acad Sci U S A.

[30] Golovkin M, Spitsin S, Andrianov V, Smirnov Y, Xiao Y (2007). Smallpox subunit vaccine produced in Planta confers protection in mice.. Proc Natl Acad Sci U S A.

[31] Lustig S, Fogg C, Whitbeck JC, Moss B (2004). Synergistic neutralizing activities of antibodies to outer membrane proteins of the two infectious forms of vaccinia virus in the presence of complement.. Virology.

[32] Xiao Y, Aldaz-Carroll L, Ortiz AM, Whitbeck JC, Alexander E (2007). A protein-based smallpox vaccine protects mice from vaccinia and ectromelia virus challenges when given as a prime and single boost.. Vaccine.

[33] Fogg CN, Americo JL, Lustig S, Huggins JW, Smith SK (2007). Adjuvant-enhanced antibody responses to recombinant proteins correlates with protection of mice and monkeys to orthopoxvirus challenges.. Vaccine.

[34] Buchman GW, Cohen ME, Xiao Y, Richardson-Harman N, Silvera P (2010). A protein-based smallpox vaccine protects non-human primates from a lethal monkeypox virus challenge.. Vaccine.

[35] Heraud JM, Edghill-Smith Y, Ayala V, Kalisz I, Parrino J (2006). Subunit recombinant vaccine protects against monkeypox.. J Immunol.

[36] Benhnia MR, McCausland MM, Su HP, Singh K, Hoffmann J (2008). Redundancy and plasticity of neutralizing antibody responses are cornerstone attributes of the human immune response to the smallpox vaccine.. J Virol.

[37] Moyron-Quiroz JE, McCausland MM, Kageyama R, Sette A, Crotty S (2009). The smallpox vaccine induces an early neutralizing IgM response.. Vaccine.

[38] Vanderplasschen A, Hollinshead M, Smith GL (1997). Antibodies against vaccinia virus do not neutralize extracellular enveloped virus but prevent virus release from infected cells and comet formation.. J Gen Virol 78 ( Pt.

[39] Bell E, Shamim M, Whitbeck JC, Sfyroera G, Lambris JD (2004). Antibodies against the extracellular enveloped virus B5R protein are mainly responsible for the EEV neutralizing capacity of vaccinia immune globulin.. Virology.

[40] Law M, Smith GL (2001). Antibody neutralization of the extracellular enveloped form of vaccinia virus.. Virology.

[41] Viner KM, Isaacs SN (2005). Activity of vaccinia virus-neutralizing antibody in the sera of smallpox vaccinees.. Microbes Infect.

[42] Benhnia MR, McCausland MM, Laudenslager J, Granger SW, Rickert S (2009). Heavily isotype-dependent protective activities of human antibodies against vaccinia virus extracellular virion antigen B5.. J Virol.

[43] Benhnia MR, McCausland MM, Moyron J, Laudenslager J, Granger S (2009). Vaccinia virus extracellular enveloped virion neutralization in vitro and protection in vivo depend on complement.. J Virol.

[44] Mehlhop E, Whitby K, Oliphant T, Marri A, Engle M (2005). Complement activation is required for induction of a protective antibody response against West Nile virus infection.. J Virol.

[45] Wang SY, Veeramani S, Racila E, Cagley J, Fritzinger DC (2009). Depletion of the C3 component of complement enhances the ability of rituximab-coated target cells to activate human NK cells and improves the efficacy of monoclonal antibody therapy in an in vivo model.. Blood.

[46] Vogel CW, Muller-Eberhard HJ (1984). Cobra venom factor: improved method for purification and biochemical characterization.. J Immunol Methods.

[47] Bengali Z, Townsley AC, Moss B (2009). Vaccinia virus strain differences in cell attachment and entry.. Virology.

[48] Ichihashi Y, Takahashi T, Oie M (1994). Identification of a vaccinia virus penetration protein.. Virology.

[49] Vanderplasschen A, Mathew E, Hollinshead M, Sim RB, Smith GL (1998). Extracellular enveloped vaccinia virus is resistant to complement because of incorporation of host complement control proteins into its envelope.. Proc Natl Acad Sci U S A.

[50] Charreau B, Cassard A, Tesson L, Le Mauff B, Navenot JM (1994). Protection of rat endothelial cells from primate complement-mediated lysis by expression of human CD59 and/or decay-accelerating factor.. Transplantation.

[51] Brazolot Millan CL, Weeratna R, Krieg AM, Siegrist CA, Davis HL (1998). CpG DNA can induce strong Th1 humoral and cell-mediated immune responses against hepatitis B surface antigen in young mice.. Proc Natl Acad Sci U S A.

[52] Chu RS, Targoni OS, Krieg AM, Lehmann PV, Harding CV (1997). CpG oligodeoxynucleotides act as adjuvants that switch on T helper 1 (Th1) immunity.. J Exp Med.

[53] Zimmermann S, Egeter O, Hausmann S, Lipford GB, Rocken M (1998). CpG oligodeoxynucleotides trigger protective and curative Th1 responses in lethal murine leishmaniasis.. J Immunol.

[54] Weeratna RD, Brazolot Millan CL, McCluskie MJ, Davis HL (2001). CpG ODN can re-direct the Th bias of established Th2 immune responses in adult and young mice.. FEMS Immunol Med Microbiol.

[55] Aldaz-Carroll L, Whitbeck JC, Ponce de Leon M, Lou H, Hirao L (2005). Epitope-mapping studies define two major neutralization sites on the vaccinia virus extracellular enveloped virus glycoprotein B5R.. J Virol.

[56] Zipfel PF, Skerka C (2009). Complement regulators and inhibitory proteins.. Nat Rev Immunol.

[57] Da Costa XJ, Brockman MA, Alicot E, Ma M, Fischer MB (1999). Humoral response to herpes simplex virus is complement-dependent.. Proc Natl Acad Sci U S A.

[58] Pozdnyakova O, Guttormsen HK, Lalani FN, Carroll MC, Kasper DL (2003). Impaired antibody response to group B streptococcal type III capsular polysaccharide in C3- and complement receptor 2-deficient mice.. J Immunol.

[59] Ochsenbein AF, Pinschewer DD, Odermatt B, Carroll MC, Hengartner H (1999). Protective T cell-independent antiviral antibody responses are dependent on complement.. J Exp Med.

[60] Panchanathan V, Chaudhri G, Karupiah G (2010). Antiviral protection following immunization correlates with humoral but not cell-mediated immunity.. Immunol Cell Biol.

[61] Panchanathan V, Chaudhri G, Karupiah G (2005). Interferon function is not required for recovery from a secondary poxvirus infection.. Proc Natl Acad Sci U S A.

[62] Panchanathan V, Chaudhri G, Karupiah G (2006). Protective immunity against secondary poxvirus infection is dependent on antibody but not on CD4 or CD8 T-cell function.. J Virol.

[63] Azeredo da Silveira S, Kikuchi S, Fossati-Jimack L, Moll T, Saito T (2002). Complement activation selectively potentiates the pathogenicity of the IgG2b and IgG3 isotypes of a high affinity anti-erythrocyte autoantibody.. J Exp Med.

[64] Ey PL, Russell-Jones GJ, Jenkin CR (1980). Isotypes of mouse IgG--I. Evidence for ‘non-complement-fixing’ IgG1 antibodies and characterization of their capacity to interfere with IgG2 sensitization of target red blood cells for lysis by complement.. Mol Immunol.

[65] Neuberger MS, Rajewsky K (1981). Activation of mouse complement by monoclonal mouse antibodies.. Eur J Immunol.

[66] Nimmerjahn F, Ravetch JV (2005). Divergent immunoglobulin g subclass activity through selective Fc receptor binding.. Science.

[67] Presta LG (2008). Molecular engineering and design of therapeutic antibodies.. Curr Opin Immunol.

[68] Hessell AJ, Hangartner L, Hunter M, Havenith CE, Beurskens FJ (2007). Fc receptor but not complement binding is important in antibody protection against HIV.. Nature.

[69] Delaney KN, Phipps JP, Johnson JB, Mizel SB (2010). A recombinant flagellin-poxvirus fusion protein vaccine elicits complement-dependent protection against respiratory challenge with vaccinia virus in mice.. Viral Immunol.

[70] Carter RH, Fearon DT (1992). CD19: lowering the threshold for antigen receptor stimulation of B lymphocytes.. Science.

[71] Fischer MB, Goerg S, Shen L, Prodeus AP, Goodnow CC (1998). Dependence of germinal center B cells on expression of CD21/CD35 for survival.. Science.

[72] Klinman DM, Klaschik S, Sato T, Tross D (2009). CpG oligonucleotides as adjuvants for vaccines targeting infectious diseases.. Adv Drug Deliv Rev.

[73] Klinman DM (2006). Adjuvant activity of CpG oligodeoxynucleotides.. Int Rev Immunol.

[74] Jouvin-Marche E, Morgado MG, Leguern C, Voegtle D, Bonhomme F (1989). The mouse Igh-1a and Igh-1b H chain constant regions are derived from two distinct isotypic genes.. Immunogenetics.

[75] Martin RM, Brady JL, Lew AM (1998). The need for IgG2c specific antiserum when isotyping antibodies from C57BL/6 and NOD mice.. J Immunol Methods.

