# Supplementary figures and images for: Antibody against Extracellular Vaccinia Virus (EV) Protects Mice through Complement and Fc Receptors
Source: PLoS One. 2011 Jun 8;6(6):e20597. doi: 10.1371/journal.pone.0020597 (PMC3110783; doi:10.1371/journal.pone.0020597)

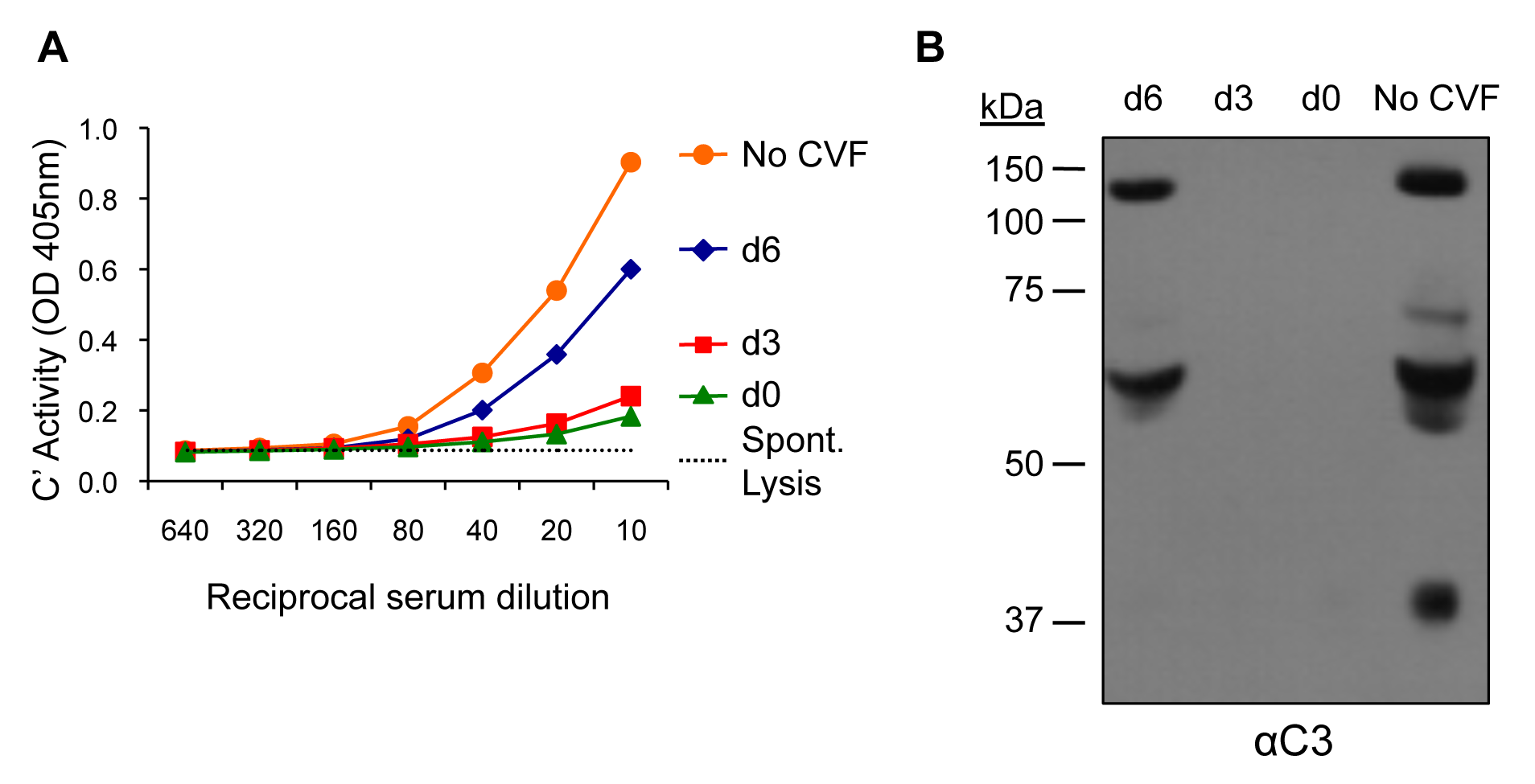

Supplement: Figure S1 — Cobra Venom Factor (CVF) treatment of BALB/c mice transiently depletes complement. To assess the degree of C' depletion after CVF treatment, groups of 11- to 12-week old female BALB/c mice (2 mice per group) were treated with CVF and then terminally bled the following day. One group was treated on day -1 and bled the next day (d0). A second group was treated on days -1 and +2 and bled the next day (d3), A third group was treated on days −1, +2, and +5 and bled the next day (d6). A group of untreated mice was used a control (No CVF). (A) CH50 assay using rabbit erythrocytes (Complement Technology, Tyler, TX) sensitized with goat anti-rabbit erythrocyte antibody (MP Biomedicals, Solon, OH) was performed with sera from mice treated or not treated with CVF. Complement activity levels on days 0 and 3 were low, while some complement activity returned by day 6. Note that sensitized rabbit erythrocytes were used because sensitized sheep erythrocytes are resistant to lysis by mouse complement. (B) Western blot of C3 protein in sera of mice treated or not treated with CVF. Serum (2 µl) from the indicated group of mice was loaded on to a 10% polyacrylamide gel. After blotting, HRP-conjugated goat anti-mouse C3 antibody (MP Biomedicals, Solon, OH) at 1∶10,000 was used to probe for the presence of C3. C3 protein was not detected on days 0 and 3, while some C3 protein was detected on day 6. (TIF) [file pone.0020597.s001.tif]

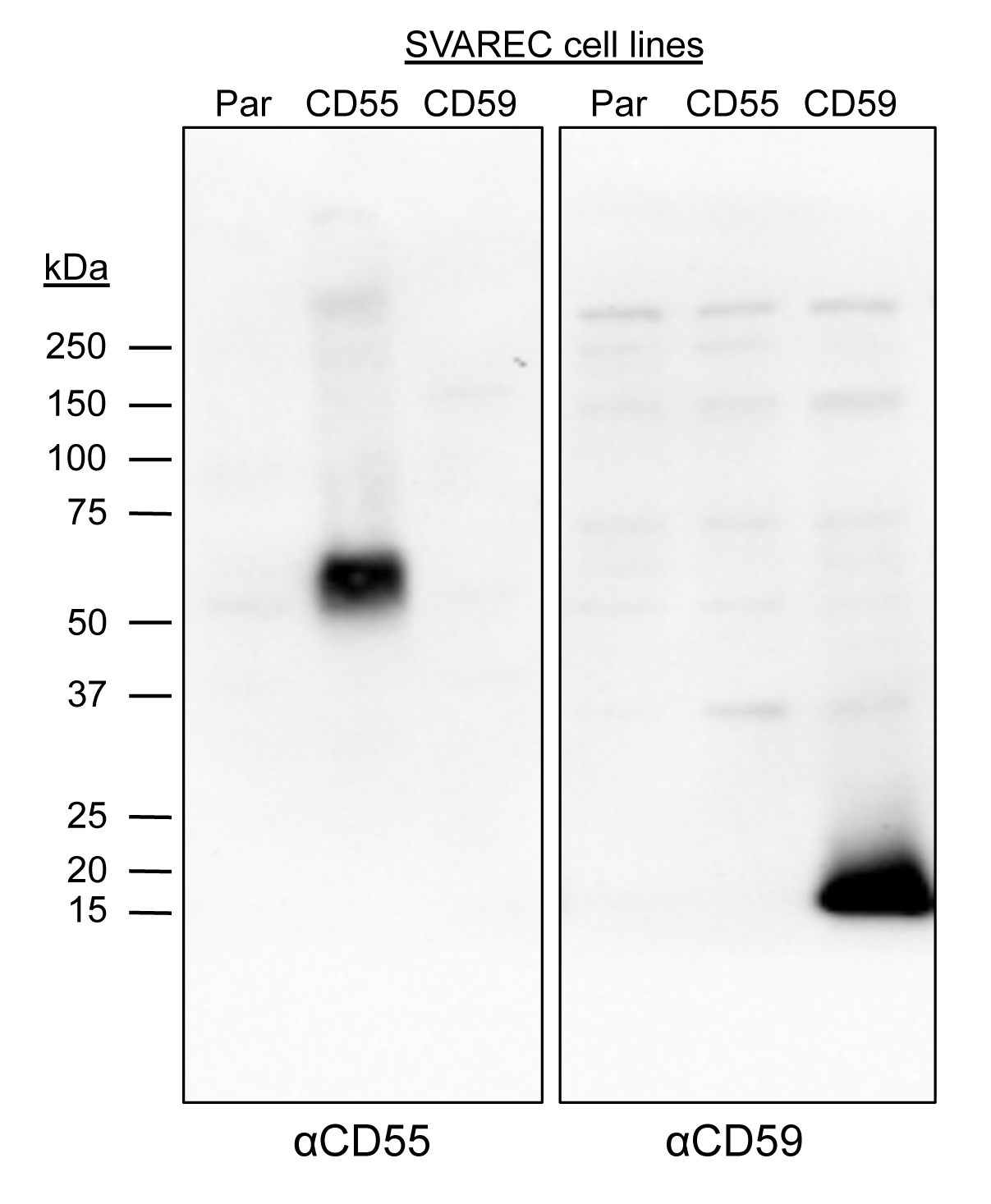

Supplement: Figure S2 — CD55 and CD59 are detected in stably transfected SVAREC cell lines. SVAREC cell lines stably transfected to express CD55 and CD59 [49], [50] were grown under selective pressure as described in materials and methods. Western blotting for either CD55 (αCD55) or CD59 (αCD55) was performed on lysates from the parental SVAREC cell line expressing no human complement regulators (Par), a SVAREC cell line expressing CD55 (CD55), and a SVAREC cell line expressing CD59 (CD59). Rabbit polyclonal anti-human CD55 and anti-human CD59 antibodies were used at a dilution of 1∶250. CD55 was only detected in the CD55-expressing cell line at its expected size of ∼70 kDa and CD59 was only detected in the CD59-expressing cell line at its expected size of ∼20 kDa. (TIF) [file pone.0020597.s002.tif]
